# Supplementary figures and images for: Efficacy of LaAg Vaccine Associated with Saponin Against Leishmania amazonensis Infection
Source: Vaccines (Basel). 2025 Jan 27;13(2):129. doi: 10.3390/vaccines13020129 (PMC11861163; doi:10.3390/vaccines13020129)

**A**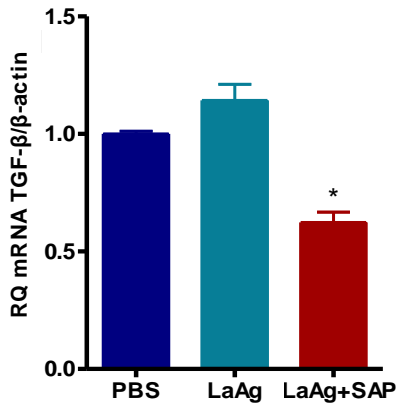**B**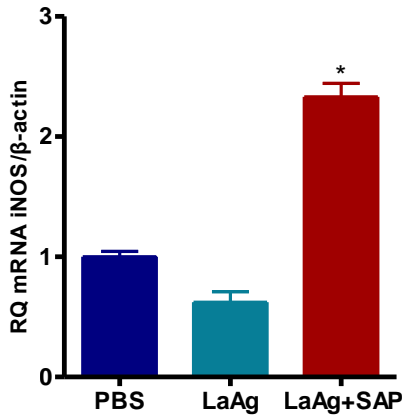

Supplement: Supplementary file 1 [file vaccines-13-00129-s001.zip › vaccines-3185473-supplementary/Supplementar/Figure S1.pdf]

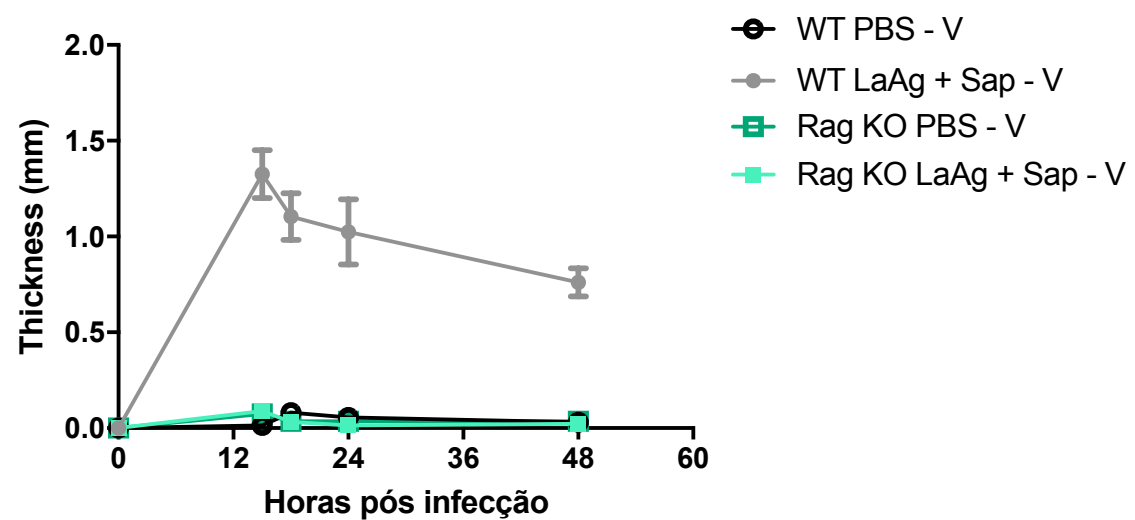

Supplement: Supplementary file 1 [file vaccines-13-00129-s001.zip › vaccines-3185473-supplementary/Supplementar/Figure S2.pdf]
